# A Sensitivity-Enhanced Vertical-Resonant MEMS Electric Field Sensor Based on TGV Technology

**DOI:** 10.3390/mi15030356

**Published:** 2024-02-29

**Authors:** Yahao Gao, Simin Peng, Xiangming Liu, Yufei Liu, Wei Zhang, Chunrong Peng, Shanhong Xia

**Affiliations:** 1 State Key Laboratory of Transducer Technology, Aerospace Information Research Institute, Chinese Academy of Sciences, Beijing 100190, China; gaoyahao18@mails.ucas.ac.cn (Y.G.); pengsimin18@mails.ucas.ac.cn (S.P.); liuxiangming18@mails.ucas.edu.cn (X.L.); liuyufei21@mails.ucas.ac.cn (Y.L.); zhangwei178@mails.ucas.ac.cn (W.Z.); crpeng@mail.ie.ac.cn (C.P.); 2 School of Electronic, Electrical and Communication Engineering, University of Chinese Academy of Sciences, Beijing 100049, China

**Keywords:** electric field sensor, MEMS, resonator, TGV technology

## Abstract

In order to enhance the sensitivity of wafer-level vacuum-packaged electric field sensors, this paper proposed a vertical-resonant MEMS electric field sensor based on TGV (Through Glass Via) technology. The microsensor is composed of the electric field sensing cover, the drive cover, and the SOI-based microstructures between them. TGV technology is innovatively used to fabricate the electric field sensing cover and the vertically-driven cover. The external electric field is concentrated and transmitted to the area below the silicon plate in the center of the electric field sensing cover through a metal plate and a metal pillar, reducing the coupling capacitance between the silicon plate and the packaging structure, thereby achieving the enhanced transmission of the electric field. The sensitivity-enhanced mechanism of the sensor is analyzed, and the key parameters of the sensor are optimized through finite element simulation. The fabrication process is designed and realized. A prototype is tested to characterize its performance. The experimental results indicate that the sensitivity of the sensor is 0.82 mV/(kV/m) within the electrostatic electric field ranging from 0–50 kV/m. The linearity of the sensor is 0.65%.

## 1. Introduction

Electrostatic field detection technology is widely used in many fields such as power grid [1,2], aerospace [3,4], meteorology [5,6], and industrial production [7,8]. In recent decades, with the development of Micro-Electro-Mechanical Systems (MEMS) technology, electric field sensors based on MEMS have become a research hotspot due to their small size, low power consumption, and mass production. MEMS-based electric field sensors can be classified into various types, such as charge induction [9,10,11,12,13], electrostatic force [14,15,16], and steered electron [17] field sensors. Resonant electric field microsensors based on charge induction have become the mainstream of MEMS electric field sensors due to their high sensitivity characterization.

In order to improve the environmental adaptability of resonant MEMS electric field sensors as well as to further improve their performance, it is necessary to package the microsensors. Currently, there are few research reports on the packaging of MEMS electric field sensors, and the existing reports mainly focus on the design and research of chip-level packaging structures. For example, in 2012, Zhang et al. [18] proposed a packaging of electric field sensors, in which a polytetrafluoroethylene (PTFE) material is used to fabricate the cap and the 14-pin dual in-line (DIP14) alloy shell is used as the substrate. The packaging avoids the electrostatic shielding of the external electric field to be measured but there are issues such as a complicated preparation process. In 2014, Wen et al. [19] proposed a packaging structure consisting of a metal cap and a polymer substrate which can protect the polymer substrate from external friction, rain, and dust, thereby effectively reducing humidity and charge accumulation. In 2015, Wu et al. [20] proposed a chip-level vacuum packaging scheme for resonant electric field microsensor, which uses ceramic eutectic bonding technology to achieve the vacuum packaging. The packaged sensor has a quality factor of 30,727.4 and a vacuum degree better than 1 Pa under driving voltages of 0.1 V DC and 0.06 V AC. However, the manufacturing process is complex, and the yield is low. In 2021, Wen et al. [21] proposed a structure for chip-level packaging of electric field sensors adopting the parallel sealing welding technique, which further improves the sensitivity of the sensor. However, there are still problems such as low manufacturing efficiency and high driving voltages. In 2022, Liu et al. [22] proposed a wafer-level vacuum-packaged electric field microsensor for the first time. The microsensor has a sensitivity of 0.16 mV/(kV/m) with driving voltages of 5 V DC and 0.05 V AC. The microsensor features a horizontal resonant type structure, and its sensitivity is not high.

Compared with horizontal resonant electric field microsensors, vertical resonant electric field microsensors can modulate the electric field more effectively, which is expected to improve the sensitivity of the electric field microsensors. Therefore, achieving a wafer-level vacuum packaging of the vertical resonant MEMS electric field sensor is meaningful. However, the key technical difficulties of wafer-level vacuum packaging for vertical resonant MEMS electric field sensors lie in the implementation of vertical driving and the construction of electric field induction channel. Wafer-level vacuum packaging technology for MEMS electric field sensors such as pressure sensors and accelerometers [23,24,25] is not suitable for electric field sensors. In 2024, Gao et al. [26] proposed a wafer-level vacuum-packaged vertical resonant electric field microsensor, in which GOS (Glass on Silicon) is used to construct the electric field induction channel. The capacitance between the electric field sensing plate and the SOI handle layer limits the sensitivity improvement of the sensor.

This paper proposed a sensitivity-enhanced vertical-resonant MEMS electric field sensor based on TGV (Through Glass Via) technology [27,28,29,30]. Taking advantage of the fact that TGV technology allows glass and silicon to exist in the same structural layer, the preparation of both an electric field sensing cover and a vertically-driven cover is achieved. The electric field sensing cover based on TGV can significantly reduce the area between the silicon plate and the SOI handle layer, thereby reducing their parasitic capacitance. It can also reduce the distance between the silicon plate and the sensitive structure at the SOI device layer. For the above reasons, the sensitivity of the sensor can be further improved.

## 2. Structure Design and Working Principle

**Figure 1:**
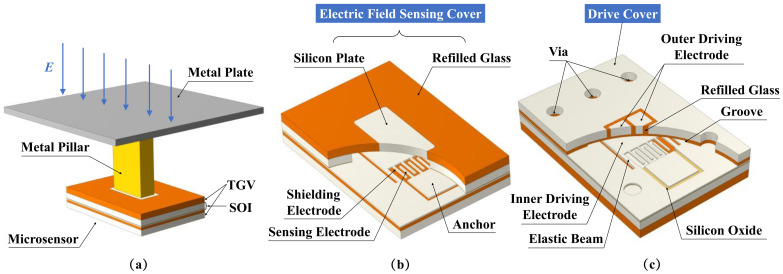
The structure diagram of the proposed electric field sensor. (**a**) The overall structure diagram of the assembled sensor. (**b**) The top view of the microsensor. (**c**) The bottom view of the microsensor.

Figure 1 shows the structure diagram of the proposed sensitivity-enhanced vertical-resonant MEMS electric field sensor based on TGV technology. As shown in Figure 1a, the overall structure is composed of the metal plate, the metal pillar, and the microsensor. The metal plate is a metal coating on the printed circuit board (PCB), which is electrically connected with the silicon plate through the metal pillar. The metal pillar lifts the microsensor to a certain height to reduce the coupling capacitance between the metal plate and the SOI handle layer.

As shown in Figure 1b,c, the microsensor consists of three parts: the electric field sensing cover, the drive cover, and the SOI-based microstructures between them. The electric field sensing cover and the drive cover are fabricated by TGV technology. The electric field sensing cover is composed of the silicon plate and the refilled glass. The silicon plate is used to transmit the external electric field to the microcavity. The refilled glass of the electric field sensing cover is used for anodic bonding as well as for reducing the parasitic capacitance between the silicon plate and SOI handle layer. The SOI microstructures consist of the shielding electrodes, the sensing electrodes, the anchors, the elastic beams, and the inner driving electrode. The drive cover is composed of the outer driving electrodes, the refilled glass, the vias, and the groove. The outer driving electrodes are two identical silicon cubes, on which two voltages with opposite signs (e.g., *V_dc_* + *V_dc_* and −*V_dc_* − *V_dc_*) are applied. This setting can not only generate the same driving force to the resonator but also eliminate the crosstalk of the driving voltages on the sensing electrodes. The refilled glass provides electric insulation between the outer driving electrodes and the silicon surrounding them. The groove provides vertical vibration space for the resonator. The electric field sensing cover, the SOI wafer, and the drive cover are anodically bonded to realize vacuum packaging.

**Figure 2:**
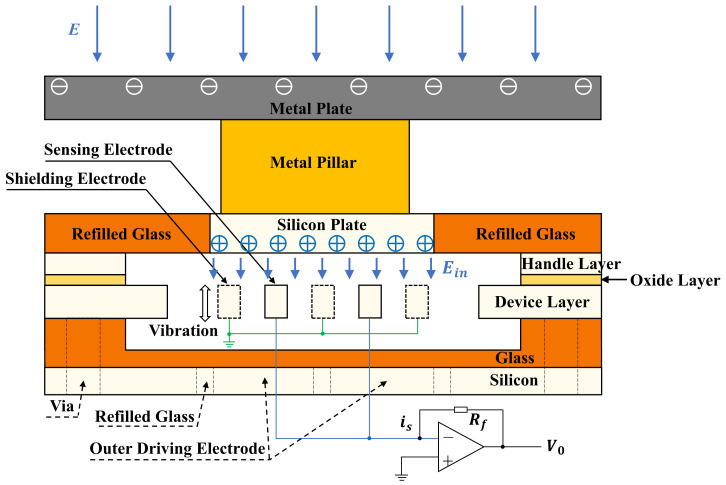
The working principle of the sensor.

The working principle of the sensor is based on charge induction. As shown in Figure 2, when there is an external electric field E, according to the Gauss theorem, a certain amount of charge will appear at the top surface of the metal plate while the opposite charge will accumulate at the lower surface of the silicon plate. The opposite charge at the lower surface of the silicon plate creates an internal electric field $E_{in}$ in the microcavity, which can be expressed as:

(1) $$E_{in}=nE$$

where n represents the scale factor, which is related to the size of the metal plate, the silicon plate, and the metal pillar. The sensing electrodes and the shielding electrodes are at the same plane when they are in the equilibrium position. According to Gauss theorem, a certain amount of charge *Q* will be induced at the sensing electrodes, which can be expressed as:

(2) $$Q=\varepsilon_{0}∯E_{in}\cdot \;dA$$

where $\varepsilon_{0}$ is the dielectric constant in vacuum, and A is the surface area of the sensing electrodes. Driven by the outer driving electrodes, the shielding electrodes that are connected to the inner driving electrode vibrate harmonically with an angular frequency of ω (ω=2πf, where f is the frequency of the AC driving voltage), which will induce periodic changes of the electric field distribution around the sensing electrodes. Thus, the induced charge $Q_{t}$ at the surface of the sensing electrodes will change periodically; it can be expressed as:

(3) $$Q_{t}=Q_{0}+Q_{s}\sin\left(\omega t\right)$$

where $Q_{0}$ is the initial charge at the sensing electrodes. $Q_{s}$ is the amplitude of the charge variation at the sensing electrodes and can be written as:

(4) $$Q_{s}=kE_{in}=nkE$$

where k represents the charge variation in the electric field of 1 kV/m, a coefficient related to the sensitive structure design and the vibration amplitude of the resonator. The changing induced charge forms an induced current at the anchors and the induced current is fed to a transimpedance amplifier to realize I-V conversion. The induced current $i_{s}$ and the output voltage $V_{0}$ can be expressed as, respectively:

(5) $$i_{s}=\frac{dQ_{t}}{dt}=n\omega kEcos\left(\omega t\right)$$

(6) $$V_{0}=i_{s}R_{f}=nR_{f}\omega kEcos\left(\omega t\right)$$

where $R_{f}$ is the gain resistance of the transimpedance amplifier. It can be seen that the output voltage $V_{0}$ is proportional to the intensity of the external electric field E for the unchanged structural parameters. By optimizing the structural parameters such that n is greater than 1 and k is as large as possible, the sensitivity of the microsensor can be enhanced.

## 3. Simulation

**Figure 3:**
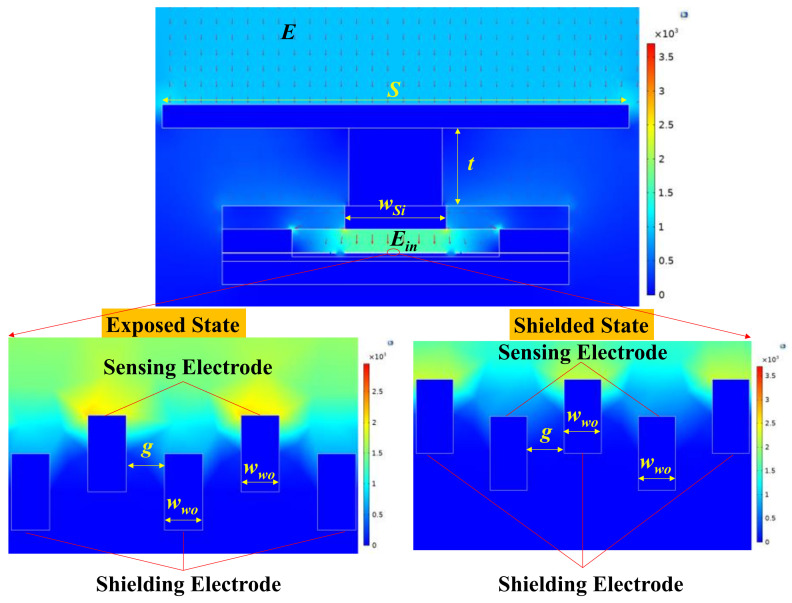
The simulation model established in COMSOL.

In this section, finite element simulations are conducted to optimize the key parameters that affect the sensitivity of the microsensor. As shown in Figure 3, a finite element structural model is established. A uniform electric field of E is applied to the metal plate and the internal electric field $E_{in}$ is generated in the microcavity, which is imposed on the sensing electrodes and the shielding electrodes (referred to as the working electrodes). The silicon plate is set to floating potential. The working electrodes, SOI handle layer, and silicon in the drive cover are grounded. The displacement of the shielding electrodes is set to 5 μm. By simulating the charge of the sensing electrodes in the exposed state and shielded state under different structural parameters, the charge variation can be calculated.

### 3.1. Optimization of the Width of the Silicon Plate

**Figure 4:**
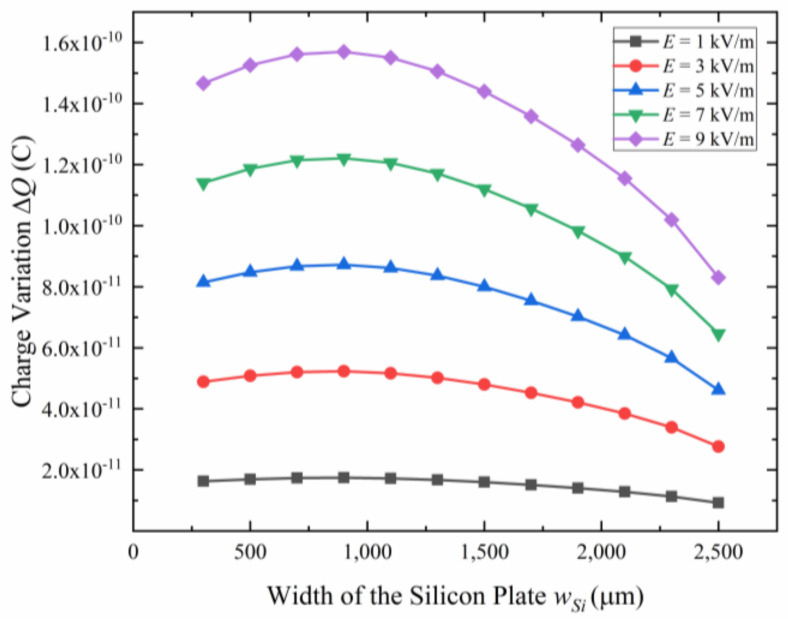
The charge variation Δ*Q* at the sensing electrodes versus the width of the silicon plate $w_{Si}$ for different electric field intensity E.

As the creator of the internal electric field $E_{in}$, the silicon plate plays an important role in the design of the microsensor. The area ratio of the silicon plate and the refilled glass decides the parasitic capacitance between the silicon plate and the SOI handle layer, which affects the sensitivity of the microsensor. Restricted by the suspended structure of the sensing electrodes and the anchors, the length of the silicon plate cannot be adjusted in a large range, so the width of the silicon plate determines its area. Keeping other parameters unchanged and changing the width of the silicon plate $w_{Si}$ simulates the charge variation of the sensing electrodes. As shown in Figure 4, with the increase of the width of the silicon plate, the charge variation of sensing electrodes increases first and then decreases. The charge variation reaches a maximum when the width of the silicon plate is 900 μm. Considering manufacturing errors such as an undercut in deep reactive ion etching (DRIE), 1000 μm is chosen as the width of the silicon plate.

### 3.2. Optimization of the Working Electrodes

**Figure 5:**
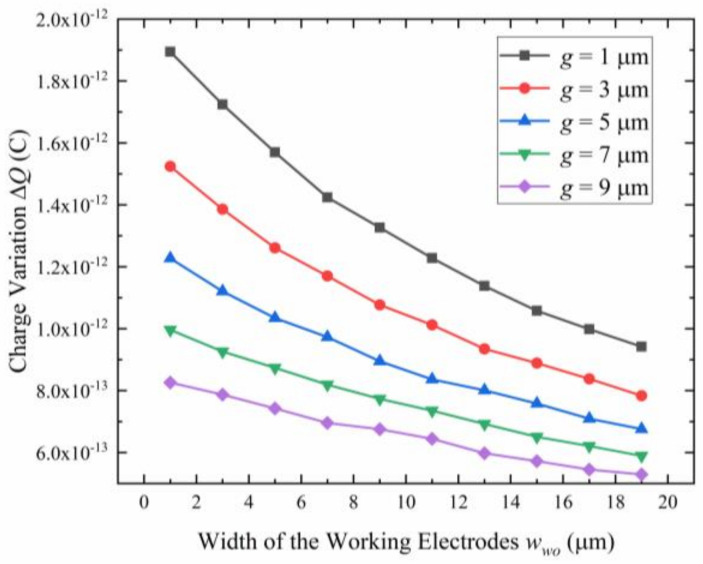
The charge variation Δ*Q* at the sensing electrodes versus the width of the working electrodes $w_{wo}$ for different gaps between two adjacent working electrodes *g*.

The size of the sensitive structure and the vibration amplitude determine the coefficient *k*, which has a significant impact on the output sensitivity of the microsensor according to Equation (6). The size of the sensitive structure mainly includes the width of the working electrodes $w_{wo}$ and the gap between two adjacent electrodes *g*. Figure 5 shows the relationship between the charge variation and the width and gap of the working electrodes. It can be seen under the same sensing area, the smaller the width of the working electrodes, the greater the charge variation. The same is true for the gap of the working electrodes. Considering the difficulty of process implementation, a width of 5 μm is chosen for both $w_{wo}$ and *g*.

### 3.3. Optimization of the Height of the Metal Pillar

**Figure 6:**
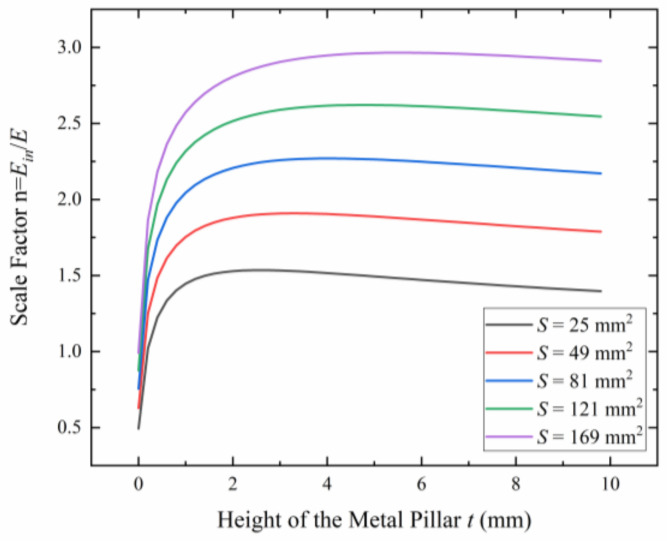
The relationship between the scale factor *n* and the height of the metal pillar *t* for different areas of the metal plate *S*.

If the metal plate is too close to the microsensor, the coupling capacitance between the metal plate and the handle layer of SOI will increase, thereby weakening the internal electric field $E_{in}$. A proper height of the metal pillar *t* can increase the scale factor *n*, which can improve the sensitivity of the microsensor. As shown in Figure 6, as the height of the metal pillar increases, the scale factor increases first and then gradually decreases. Considering the actual operational difficulty, a 2 mm height metal pillar is selected.

The key parameters of the electric field sensor are shown in Table 1.

## 4. Device Fabrication

**Figure 7:**
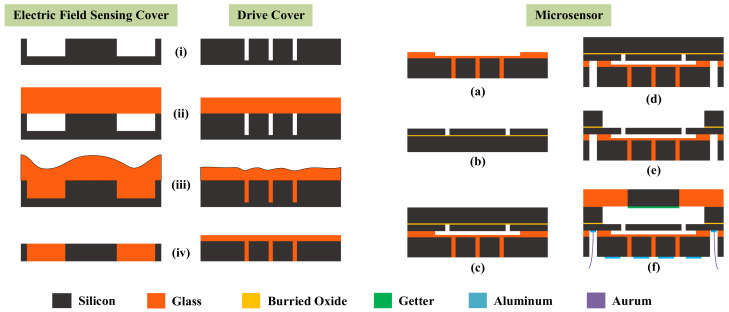
The fabrication process of the TGV wafers (**i**–**iv**) and the microsensor (**a**–**f**).

As shown in Figure 7, the fabrication process of the microsensor is based on TGV technology and bulk micromachining, which can be divided into the preparation of the TGV wafers and the fabrication of the microsensor.

### 4.1. Preparation of the TGV Wafers

The electric field sensing cover and the drive cover are fabricated by TGV technology. The key fabrication process of the two TGV wafers is shown in Figure 7i–iv. The detailed steps are described as follow:

(i) A 530 μm thick highly-doped silicon wafer is etched 320 μm deep in an AMS-100 DRIE (Deep Reactive Ion Etching) system produced by Alcatel from France, forming a mold. A 390 μm thick highly-doped silicon wafer is etched 330 μm deep in an Omega Lpx Rapier DRIE system produced by SPTS Technologies Ltd. from Britain.
(ii) The etched silicon wafers are anodically bonded to a 500 μm thick borosilicate glass (BF33) wafer and a 300 μm thick BF33 glass wafer, respectively, in vacuum conditions using a SB6e bonder produced by SUSS MicroTec from Germany at voltages of 1000 V and 800 V, respectively, with a pressure of 800 mBar.
(iii) The bonded wafer is heated in a muffle furnace produced by FNS Electric Furnace Co., Ltd. from China (900 °C for 5 h) to reflow the glass into the silicon mold under atmospheric pressure.
(iv) The reflowed glass surface and the back surface are chemically and mechanically polished (CMP) using a GNAD61 precision grinding and polishing machine produced by FEE Company from Germany. The total thickness variation (TTV) of the wafer after CMP is within 5 μm and the roughness of the wafer after CMP is less than 50 Å, which fully meets the requirements of anodic bonding.

### 4.2. Fabrication Process of the Microsensor

The microsensor is fabricated using the bulk micromachining process as shown in Figure 7a–f. The detailed steps are described as follows:

(a) The hydrofluoric acid vapor is utilized to corrode a 30 μm deep groove at the glass layer of the drive cover, which is used to provide the vertical vibration space for the resonator.
(b) The device layer of SOI is etched in a HSE M200 DRIE system produced by NAURA Company from China by which the resonator, the sensing electrodes, and the anchors are patterned.
(c) The SOI wafer and the drive cover are anodically bonded in vacuum conditions using an SUSS SB6e bonder at a voltage of 430 V with a pressure of 400 mBar.
(d) DRIE and hydrofluoric acid vapor are used successively to etch the holes used for lead wires. DRIE is conducted in a NAURA HSE M200 DRIE system.
(e) DRIE is used to etch the handle layer of SOI to form the vibration cavity and RIE (Reactive Ion Etching) is used to etch the buried oxide layer of SOI to release the movable structure in the NAURA HSE M200 DRIE system.
(f) The getter (1 μm thick Ti) is deposited on the surface of the silicon plate using a BJD-2000 electron beam evaporation system produced by Ferrotec from Japan. The electric field sensing cover and the released wafer are anodically bonded in the SUSS SB6e bonder at a voltage of 800 V with a pressure of 400 mBar. Finally, 1 μm thick aluminum pads are deposited on the areas that need lead wires in a Ferrotec BJD-2000 electron beam evaporation system.

**Figure 8:**
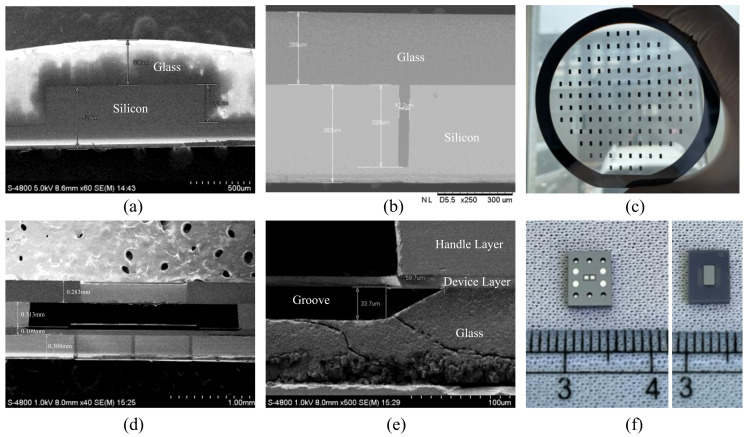
The experimental photos in the fabrication process. (**a**) Cross-sectional SEM photo of the electric field sensing cover after the glass reflow process. (**b**) Cross-sectional SEM photo of the drive cover after glass reflow process. (**c**) Photo of the electric field sensing cover after CMP. (**d**) Cross-sectional SEM photo of the microsensor. (**e**) Cross-sectional SEM photo of the area near the anchor. (**f**) Front and back photos of the microsensor after dicing.

The key experimental photos are shown in Figure 8. Figure 8a,b show the cross-sectional SEM photos of the two TGV wafers after the glass reflow process. In Figure 8a, the gray area is glass and the black area is silicon. It can be seen that the silicon wafer is 529 μm thick and the etched groove is 321 μm deep and is completely filled with glass. In Figure 8b, the dark gray area is glass and the light gray area is silicon. The width of the etched deep groove is 42.2 μm, which is basically consistent with the design value of 40 μm. The difference is caused by photolithography errors and etching errors. It can be seen that the 330 μm deep silicon groove is completely filled with glass. Therefore, the glass filling rate of both of the two TGV wafers reached 100%. Figure 8c shows the photo of the TGV wafer used as the electric field sensing plate after CMP. Figure 8d shows the cross-sectional SEM photo of the microsensor, in which the sandwich structure, including the electric field sensing cover, SOI, and the drive cover, can be clearly distinguished. The dark gray area is glass and the light gray area is silicon. It can be seen that the thickness of the glass layer of the drive cover after CMP is 0.109 mm, which is basically consistent with the design value of 100 μm. Figure 8e shows the cross-sectional SEM photo near the anchor. It can be seen that the depth of the groove is 33.7 μm, which agrees with the designed value of 30 μm. The lateral corrosion by HF is 59.7 μm, and it can be inferred that the ratio of hydrofluoric acid corrosion depth to side drilling is about 1:2. Figure 8f shows the front and back photos of the microsensor after dicing. The overall size of the microsensor is 5.4 mm × 6.6 mm × 1.0 mm.

## 5. Experiment

### 5.1. Experiment Setup

**Figure 9:**
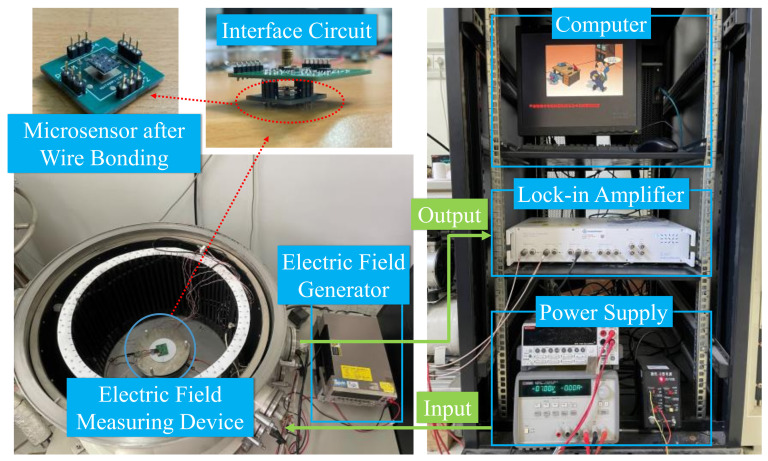
Photos of the testing system.

As shown in Figure 9, the test system is composed of a computer, a lock-in amplifier, power supplies, an electric field generator, and an electric field measuring device. The power supplies provide DC voltage for the interface circuit and the microsensor. The microsensor is fixed at the gold layer of the PCB through a copper pillar, in which the gold layer is connected to the gold layer on the back of the PCB through a small hole in the middle of the PCB. The wire bonding is then conducted.

**Figure 10:**
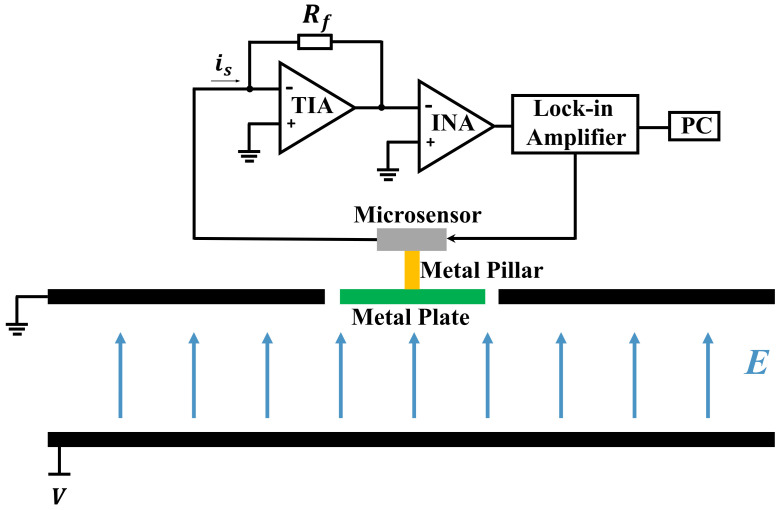
Schematic of the electric field testing.

The principle of the electric field test is shown in Figure 10. It can be seen that the standard electrostatic field is generated by two parallel metal plates supported by three polytetrafluoroethylene columns, where the upper plate is grounded while the potential of the lower plate is *V*. The area of both of the metal plates is 314 cm^2^ and the gap between them is 2 cm. There is a square hole with a side length of 2.5 cm in the center of the upper plate, in which the metal plate can be placed. The voltage applied to the parallel metal plates is generated by the Keithley 2400 high voltage source meter, which has an accuracy of 0.012%. At a maximum output voltage of 1000 V, the minimum resolution of the output voltage is 0.12 V. In other words, the minimum resolution of the generated electric field is 6 V/m. The AC drive voltage for the microsensor is provided by the lock-in amplifier. The interface circuit mixes DC and AC driving voltages and applies them to the outer driving electrodes of the microsensor. In addition, the interface circuit converts the output current signal into a voltage signal, which is then fed to an instrumental amplifier. The signal from the interface circuit is sent to the HF2LI lock-in amplifier for detection and the result is displayed on the personal computer. The minimum resolution of the HF2LI lock-in amplifier is 61 nV and in practical tests it is 61 μV at a full input voltage of 1 V.

### 5.2. Experimental Results

#### 5.2.1. Amplitude–Frequency Characteristics

**Figure 11:**
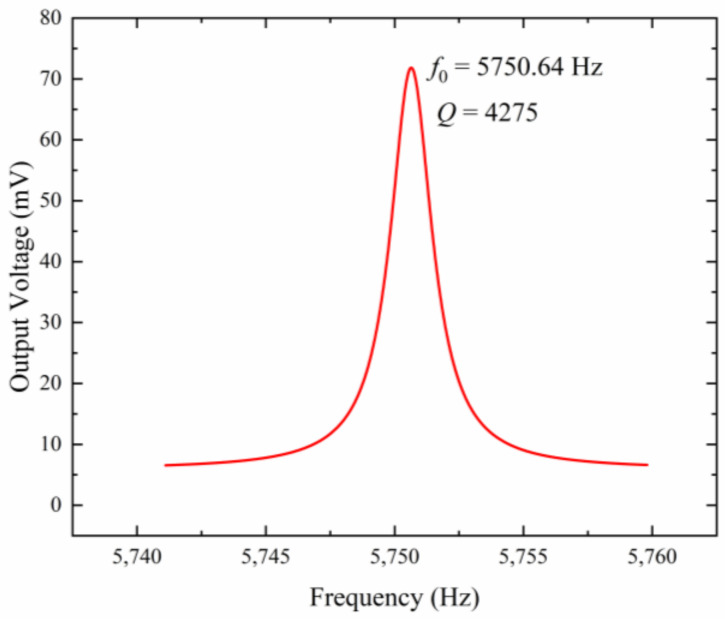
Amplitude–frequency characteristics of the microsensor.

The amplitude–frequency response of the microsensor is shown in Figure 11. The resonant frequency of the microsensor is 5750.64 Hz, which is in accordance with the simulation result of 5681.4 Hz. The discrepancy is mainly caused by the fabrication process error and the simulation deviation. The function of the amplitude–frequency response curve after Lorentz fitting can be expressed as:

(7) $$y=y_{0}+\frac{2A}{\pi }\frac{w}{4{\left(\omega -\omega_{c}\right)}^{2}+w^{2}}$$

where $y_{0}=5.857,A=203.769,w=1.966,and\;\omega_{c}=5750.64$. The 3 dB bandwidth can be calculated as Δω=1.3453 Hz.

Therefore, the quality factor of the proposed microsensor can be calculated as:

(8) $$Q=\frac{\omega_{c}}{\Delta \omega }\approx 4275$$

#### 5.2.2. Sensitivity Characteristics

**Figure 12:**
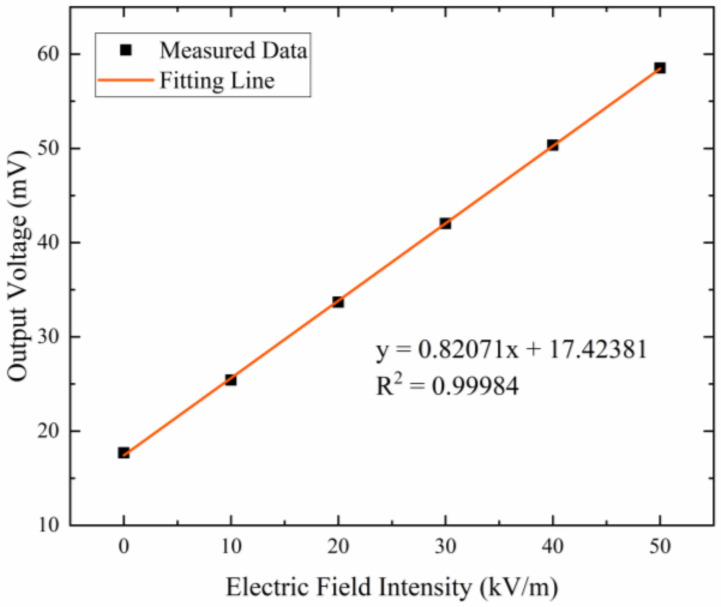
Response of the microsensor to the external electric field intensity.

Figure 12 shows the response of the microsensor to the applied electric field intensity when the microsensor works in the resonant mode. The microsensor is tested in the electric field ranging from 0–50 kV/m and the experimental result shows that the sensitivity of the microsensor is 0.82 mV/(kV/m). The linearity of the microsensor is calculated to be 0.65%. In addition, experimental results show that a driving voltage of 7 V DC and 0.07 V AC is required for the microsensor, which is beneficial to the reduction of power consumption.

#### 5.2.3. Quality Factor Test

**Figure 13:**
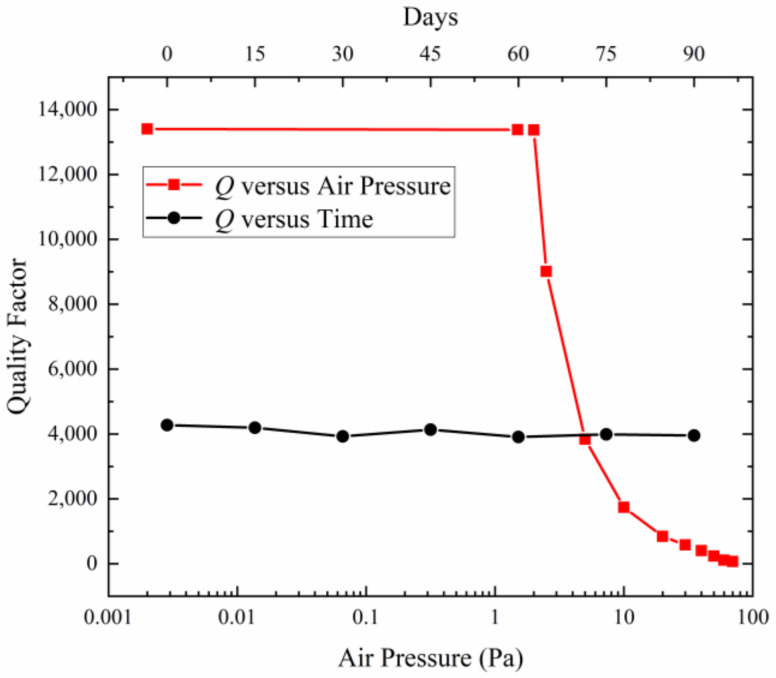
Quality factor variation over time and the relationship between quality factor and air pressure.

As shown in Figure 13, the quality factor of the microsensor is tested every 15 days. It can be seen that the quality factor remained basically around 4000 during the 90-day testing time, which indicates the airtightness of the microsensor is good.

In addition, we test the uncovered microsensor in a vacuum-controlled chamber and the relationship between the quality factor and air pressure is obtained as shown in Figure 13. It can be indicated that the packaged air pressure of the microsensor is about 5 Pa. The main factor affecting the quality factor of the microsensor is air pressure, and the quality factor can be increased by improving the fabrication process such as using the stronger getter in the future work.

## 6. Discussion

The key performance comparison of the existing wafer-level vacuum-packaged electric field sensors (WLVPEFS) is shown in Table 2. It can be seen that, compared with the existing WLVPEFS, the sensitivity and linearity of the microsensor proposed in this paper are further improved.

## 7. Conclusions

In this paper, a sensitivity-enhanced vertical-resonant MEMS electric field sensor based on TGV Technology is proposed. TGV technology is innovatively used to fabricate the electric field sensing cover and the drive cover of the microsensor. A device prototype is tested and the experimental results show that the sensitivity is 0.82 mV/(kV/m) and the linearity is 0.65% within the electrostatic electric field ranging from 0–50 kV/m. Compared with the previously reported wafer-level vacuum-packaged electric field sensors, the sensitivity and linearity in this paper have been further improved.

## Figures and Tables

**Table 1:** Key structural parameters of the electric field sensor.

Key Structural Parameters	Value
size of the silicon plate	2500 μm × 1000 μm
size of the working electrodes	630 μm × 5 μm
gap between two adjacent working electrodes	5 μm
number of the working electrodes	130 × 2
size of the elastic beams	650 μm × 50 μm
thickness of the device layer	10 μm
height of the metal pillar	2 mm
size of the inner driving electrode	1600 μm × 1100 μm
size of the outer driving electrodes	690 μm × 620 μm
width of the refilled glass at the drive cover	40 μm
thickness of the glass layer of the drive cover	100 μm
depth of the groove at the glass layer of the drive cover	30 μm
diameter of the via	670 μm

**Table 2:** Key performance comparison of the existing WLVPEFS.

Existing WLVPEFS	Structure Type	Sensitivity (mV/(kV/m))	Linearity	Quality Factor
Liu [22]	horizontal	0.16	1.62%	5738
Gao [26]	vertical	0.31	5.84%	5071
this paper	vertical	0.82	0.65%	4275

## Data Availability

Data are contained within the article.
